# Ambiguity in a masculine world: Being a BRCA1/2 mutation carrier and a man with prostate cancer

**DOI:** 10.1002/pon.4530

**Published:** 2017-09-18

**Authors:** C. Moynihan, E.K. Bancroft, A. Mitra, A. Ardern‐Jones, E. Castro, E.C. Page, R.A. Eeles

**Affiliations:** ^1^ The Institute of Cancer Research London UK; ^2^ The Royal Marsden NHS Foundation Trust London UK; ^3^ University College Hospitals London UK; ^4^ Spanish National Cancer Research Centre (CNIO) Madrid Spain

**Keywords:** *BRCA1*, *BRCA2*, gender/masculinity, prostate cancer, psychosocial

## Abstract

**Objective:**

Increased risk of prostate cancer (PCa) is observed in men with BRCA1/BRCA2 mutations. Sex and gender are key determinants of health and disease although unequal care exists between the sexes. Stereotypical male attitudes are shown to lead to poor health outcomes.

**Methods:**

Men with BRCA1/2 mutations and diagnosed with PCa were identified and invited to participate in a qualitative interview study. Data were analysed using a framework approach. “Masculinity theory” was used to report the impact of having both a BRCA1/2 mutation and PCa.

**Results:**

Eleven of 15 eligible men were interviewed. The umbrella concept of “Ambiguity in a Masculine World” was evident. Men's responses often matched those of women in a genetic context. Men's BRCA experience was described, as “on the back burner” but “a bonus” enabling familial detection and early diagnosis of PCa. Embodiment of PCa took precedence as men revealed stereotypical “ideal” masculine responses such as stoicism and control while creating new “masculinities” when faced with the vicissitudes of having 2 gendered conditions.

**Conclusion:**

Health workers are urged to take a reflexive approach, void of masculine ideals, a belief in which obfuscates men's experience. Research is required regarding men's support needs in the name of equality of care.

## 1 BACKGROUND

Men with *BRCA1/2* mutations have an increased risk of prostate cancer (PCa)^1^ with an estimated relative risk of 1.8 to 4.5 fold for *BRCA1* and 2.5 to 8.6 fold for *BRCA2* mutation carriers.^2^, ^3^ Numbers of men with PCa attributed to *BRCA1* or *BRCA2* is relatively small but rising (approximately 2% of men diagnosed under the age of 55).^1^ This is clinically important as men with *BRCA2* mutations present with aggressive PCa at a younger age and have poor survival.^4^ Awareness of PCa risk is increasing, becoming a standard part of genetic counselling for men at risk of inheriting a *BRCA1/2* mutation.

Research on women with cancer predominates^5^ forming templates for investigation premised on emotional expression and helplessness/ hopelessness^5^, ^6^ and a need for support.^7^ Men are depicted in opposite ways.^5^, ^8^, ^9^ Unequal services persist.^5^, ^6^, ^7^, ^8^, ^9^, ^10^, ^11^, ^12^ Male studies are growing especially in relation to PCa where psychosocial outcomes reflect stage, treatments received, and physical status, usually sexual “dysfunction” including impotency.^13^ When “masculinity” theory and a qualitative methodology are invoked,^12^ masculinity is restored^13^, ^14^, ^15^, ^16^ while, for example, men put impotency into perspective,^13^, ^15^ in terms of age, a trade‐off for living longer, previous “sowing oats,” and ways of compensating for penetrative sex.^13^, ^15^ Feelings of loss are, however, profound^13^, ^15^; identity, self‐esteem, coping, and adjustment issues prevail^13^, ^14^, ^15^, ^16^, ^17^, ^18^, ^19^ although levels of clinical psychological morbidity are low.^10^, ^13^, ^17^

Men with breast cancer experience stigmatisation, embarrassment, and altered body image, exacerbating their shock at diagnosis.^20^, ^21^ Formal support programmes are lacking, information is sparse, and deliverance gendered.^20^, ^21^

Both sexes at high‐risk of *BRCA1/2* are similar in their responses regarding genetic risk.^17^ Both use family histories to evaluate risk and decision‐making^22^, ^23^ although women are likely to influence male decisions.^22^ A lower uptake of testing and higher drop out in men^12^, ^17^ is counterbalanced by a greater interest in testing.^17^ Men are thought to be more vulnerable to psychological stress than women when undergoing testing^17^ although low levels of psychological symptoms are reported in both sexes undergoing testing for *BRCA1/2* mutations.^17^, ^24^

A gender approach is invisible notwithstanding a few exceptions where it is used as a backdrop to men's responses.^12^, ^18^, ^19^, ^25^ No genetic study has used “masculinity theory” as an integral aspect of research, yet sex and gender are key determinants of health.^11^

### 1.1 Masculinity theory

Psychosocial research relies on an assumption that essentialist “traits” describe men's responses. Western societies hold to “ideal” or “hegemonic” male characteristics such as stoicism, independence, control, and emotional inexpressiveness,^8^, ^9^, ^14^, ^26^, ^27^, ^28^, ^29^ while women exhibit binary opposites.^8^, ^9^, ^26^, ^27^, ^28^, ^29^ The “hegemonic,” dominant form of masculinity is always relational—subordinating femininities and other masculinities,^8^, ^9^, ^14^, ^15^, ^16^, ^26^, ^27^, ^28^, ^29^ played out individually, and in institutions^5^, ^8^, ^9^, ^23^, ^24^, ^25^, ^26^, ^27^, ^28^, ^29^ reinforcing expected responses.^8^, ^9^ Concern regarding the “fixed” nature of essentialism has resulted in research that turns towards a “social constructivist” perspective on gender or “masculinity,” the latter contingent on time and place.^8^, ^9^, ^26^, ^27^ For example, illness may undermine men in terms of loss of control and dependency, leading to new ways of reinstating masculinity or “masculinities.”^8^, ^9^, ^26^, ^27^, ^28^, ^29^ “Masculinity must be proved and no sooner proved that it is again questioned and must be proved again.”^27(p122)^

### 1.2 Aims

The experiences of male *BRCA1/2* carriers who have prostate cancer are not reported. The aim of this study was to highlight men's social characteristics to explain behaviour and attitudes where both conditions have gendered connotations by using masculinity theory^8^, ^9^, ^26^, ^27^, ^28^, ^29^ and empirical work.^10^, ^12^, ^13^, ^18^, ^19^, ^20^, ^21^, ^22^, ^25^, ^30^, ^31^, ^32^

## 2 STUDY DESIGN

The Royal Marsden NHS Research Ethics Committee approved this study. Men were identified from one UK Cancer Genetics research clinic over 4 years (2007‐2011). The clinic managed patients known to have a mutation in a cancer predisposition gene.

Eligibility relied on a diagnosis of PCa and a pathogenic germline mutation in either *BRCA1* or *BRCA2*. All patients were “counselled” regarding their disease status. Eligible patients were invited to undergo a semistructured interview, given an information sheet and reply slip to express interest. Interested men were contacted, interviews arranged, consent obtained. One‐ to two‐hour interviews were conducted in a venue of the patients' choice. Interviews were audio recorded, transcribed, and analysed. Interviewees were at liberty to stall or curtail interviews.

A female sociologist outside the clinical team, with relevant experience, asked, “Has *BRCA* mutation status and having PCa impacted on your life?” Topics informed by interviewees, clinical practice, and literature were covered flexibly (Table S1).^20^, ^21^

### 2.1 Method of analyses

A “Framework Analysis”^33^ (allowing for the use of relevant topics of interest) was used to code items at face value, followed by conceptual coding identifying overarching themes and “deviant” accounts.^33^, ^34^ Interviewing and analysis was an iterative process. Constant comparison was used; interpretations made by checking patients' perceptions within and across verbatim transcripts. Interpretation relied on context, literature, field notes, and expert opinion.^33^, ^34^

## 3 RESULTS

### 3.1 Sample

Twenty‐nine participants (11 men, 9 partners, and 9 children) were interviewed. This analysis draws on the interview data of 11 men. Two partners were present but remained silent.

Fifteen eligible men were identified from the database; 13 men accepted; one later declined; one was ineligible. Most men had children (Table S2).

Participant numbers were proportional to national figures, saturation achieved. Mean time between undergoing genetic testing and PCa diagnosis was 26.5 months. Mean time between genetic testing and study participation was 37 months. One man received his PCa diagnosis with advanced disease prior to mutation identification. The other 10 men had early stage disease. Nine men were diagnosed with PCa through PSA screening, 8 of whom were screened within a research study; 2 men were diagnosed after presenting with symptoms.

There were no discernible demographic or social differences in responses (Table S2).

### 3.2 Themes

The umbrella theme “Ambiguity in a Masculine World” wove its way through men's responses. Four subthemes were evident. Shock at carrier status or having PCa (Storm Clouds) was juxtaposed by the “bonus” of early diagnoses (Silver Linings), denial and fatalism (Brushing under Carpets) by guilt and responsibility (Facing the Music). Vulnerability and a so‐called feminine need for “attachment” were juxtaposed by a stereotypical “masculine” response of self‐determination, stoicism, control, and “normality.”

**Nigel**: “I screen for everything…I go to medical professionals and appreciate care I get from them.....BRCA is not my fault… a genetic accident. Could be worse…a genetic fault (to be) a serial murderer. I was more upset about prostate cancer…I was healthy ( ) I've researched the prostate fanatically… cancer the enemy, I the “General”....proactive…I am not so worried when I know how the enemy acts…many men don't want to know… I do, with a stoic acceptance. I might be called ‘bloody useless’ in a new relationship but I'm not a lesser person…I am still breathing, I drink wine, new hobbies!”

#### 3.2.1 Storm Clouds: the BRCA mutation

Participants were initially reluctant to test, claiming “low risk” (or no risk), a “hazy” perception of hereditary transmission and the gendering of breast cancer.^21^ No reference was made regarding the risk of ovarian cancer associated with the *BRCA1/2* genes. Far from being coerced to test,^17^ men felt a need to pay back “work” of family members:

**Rupert**: “My sister had breast cancer…MASSIVE in the family……I normally wouldn't test...but she worked hard for us…so I went, I don't go to doctors...I'm a busy man… women get the disease and everything in pamphlets is said towards them!”

Familial images of illness and death evoked the importance of testing in both sexes.^23^, ^24^ Roger expressed shock^21^, ^22^ acceptance and pragmatism:

**Roger:** “After the initial numbing shock (BRCA) I thought I'd die, I wasn't surprised... being twelve... mum's in hospital waving from her window bed…a big impact...looking Belson like…( ) I worried…what if I've got it and pass it on? I tested...I could tell my daughters.”

While “difficulties” were downplayed, a “betwixt and between” uncertainty^29^ was evident, highlighting loss of self‐esteem and disruption usually wrapped in reminders of achievement and good health both past and present.^10^

**Jeff:** “this genetic fault means imperfection…it didn't have an illness…(I was) damaged, imperfect at a time when I was healthy, working… had to re‐charge …having BRCA2 was important…had I known before meeting (wife), how would I have told someone? What if you wanted a family?....I'm completely back to myself”

A detailed account of having PCa overshadowed the telling of what it meant to have a *BRCA1/2* mutation.

#### 3.2.2 Storm Clouds: prostate cancer

Men with PCa focus on its physical aspects.^10^ Diagnosis, treatment, and side effects loomed large in our participant's accounts. Constant self‐referral wrapped itself around stoicism, optimism, and self‐determination.^10^, ^13^, ^14^, ^15^, ^16^ Distress was most profound amongst younger participants who referred to longevity; older men described the ramifications of treatment, any “crisis” normalised,^10^ all experiences perceived to be supported by partners and hospital personnel while containing emotions.^10^

**Jeff**: “ten (biopsy) cores plus two...bleeding and had to have a pad....I was thinking ‘death...get on with it.’.. had the prostatectomy...impotency and incontinence didn't matter…but I couldn't have done this without (doctor)...she guided me, was there for me...brilliant”

**Martin:** “My wife helped me through and through along with the hospital staff… Incontinence was very difficult but it is all back to normal”

The “double whammy” was seldom addressed, and when it was, prostate cancer took precedence.

**Edward:**“I was probably more upset about prostate cancer although I didn't show it…I've thrown the dice, got the double whammy… get shot of it, get on with life.”

**Nick:** “I take the view that I would prefer to deal with prostate cancer than any others because I've heard that it's more likely treated successfully if caught early....”

Treatment effects and fear of relapse, responses that did not apparently warrant formal support, were stoically presented.^9^, ^10^ “Counselling” might provide knowledge and advice, never to express emotions. When emotion was articulated, it was defended:

**George**: “After prostate cancer (PCa) surgery, I threatened to throw myself from the window...a counsellor came…it was nice having a chat…breaking up days…he left realising I wasn't mad…just reacting to the situation.”

#### 3.2.3 Silver Linings: the BRCA mutation

Shock and repercussions of the *BRCA* mutation were accompanied by a sense of “bonus.” Like women, men appreciated early recognition of *BRCA* as a preventative measure, not for themselves but for gaining knowledge and scientific progress benefitting mankind, mainly daughters.^17^, ^19^, ^25^, ^30^, ^31^, ^32^

**Roger**: “We are terribly lucky to have found out (about the gene) and it offered solutions…knowledge is power…if I'd got it…my daughter could find out too and do something”

Reproductive options were seldom mentioned, although Edward, who distanced himself from family problems, broached the subject. His concerns counter the suggestion that emotional distancing necessarily leads to downplaying risk.^17^

**Edward**: “if (son) were to start a family……they would do some in‐vitro fertilisation to check whether the gene was present… they could selectively abort… but I asked whether there was evidence of positive aspects of the BRCA1 gene… (Could) you lose that if you bred it out of the gene pool’? Answer was ‘no’!”

Like affected women,^12^, ^17^ men did not report clinical psychological impairment. Men's mutation status caused distress in terms of children, however. This is evident amongst affected women and men in other studies.^12^, ^17^, ^19^, ^22^, ^30^, ^31^, ^32^

**Martin**: “to think that our daughter might have to make a decision! It's harder worrying that (daughter) had it rather than myself”

Men's sadness at passing on the mutation was sometimes visceral but suppressed.

**Rupert**: “we love our children... in that respect one feels responsibility...I said to (daughter)...”You've got the wrong Daddy”....sorry I'll stop.” *(crying)*

Despite occasional lapses into pathos and ongoing distress, men remained optimistic “putting this *BRCA* stuff on the back burner.”

#### 3.2.4 Silver Linings: prostate cancer

The *BRCA* mutation led to concern for others.^19^, ^22^, ^25^, ^31^, ^32^ In contrast, PCa narratives manifested highly subjective accounts.^10^ If PCa was intrinsically worrying, there was a strong sense of “bonus”—care through monitoring and early diagnosis.

**Richard**: “I was delighted by the research ...the way we were individually looked after…having regular checks... if (PCa) was going to happen it would be found…multiple bonus… This program saved me…I get on with my busy life and keep on top of things.”

Clinical psychological symptoms were absent,^10^, ^12^ and formal support seldom offered or accessed.^10^, ^13^ Men showed no hesitation in rallying health providers, seeking information, and gaining knowledge.^13^, ^35^ Appreciation was continuously shown for personal aspects of supportive care received from medical personnel, the rudiments of “attachment” emphasised.^36^

**Nick**: “the doctors are brilliant…wonderful… (Nurse) was magnificent…you can ring her up, she helps you… knows you…just what I needed…I think (nurse) likes me…they really looked after you.”

#### 3.2.5 Brushing under Carpets: the BRCA mutation

Men put *BRCA* on the “back burner” despite the importance of alerting family members. Few men recounted *BRCA1/2* status, its virulence never addressed, and its low risk status in men reiterated and/or gendered. Addressing the consequences of having the mutation was overshadowed by a PCa discourse—the embodied disease.

**Jeff**: “BRCA2?… it doesn't have much impact (on me)… being male it wasn't significant… it is the prostate that worries me.”

#### 3.2.6 Brushing under Carpets: prostate cancer

While PCa narratives held sway,^10^ disclosure rarely went beyond close family and friends.^10^, ^13^, ^30^, ^37^ Men chose their confidants with care, withholding details, maintaining normality, strength, and activity in the face of shame.^10^, ^37^ A reticence to “speak” out is found amongst women in the genetic context^24^ and in men with PCa.^37^

**Nick**: “I've always been strong… eradicate the cancer…and get on ( )…Prostate cancer…shattering…… life goes on…I don't speak about it...shaming...my sex life is not as it was…it doesn't matter, I have three children!...( ) people don't want to hear...I speak to members of my church about life and death!”

“Holding back” mirrored the ways men felt about informing children of PCa claiming that knowledge would “add to their burden.” There was evidence, however, that men did not have the words to say or when to say them mirroring problems in the male *BRCA* arena.^19^, ^30^

**Jeff**: “how do I tell (children) and when? That's not today's problem...( )...I haven't thought how to tell my son...”

#### 3.2.7 Facing the Music: the BRCA mutation

Studies have shown that in contrast to men's apparent reluctance to exchange information, and the ambiguity men display in terms of transmitting genetic information to family members,^17^ women become the “gatekeepers of health,”^17^, ^24^, ^30^ resulting in the “gendering of responsibility.”^19^, ^30^, ^31^ In contrast, our male participants transmitted *BRCA1/2* information to family including female children,^17^, ^19^, ^24^, ^25^, ^32^ their fervour underpinned by guilt, and responsibility^12^, ^31^ while taking on a head of family status:

**Richard:** “I've written letters to ALL family members...through the BRCA gene…there is nothing in common except this important familial relationship… I should take on an ‘elder role’!”

Fatalism sometimes overshadowed responsibility and guilt.^31^ Multiple genes and or a simple mutation status was argued for, as disclosure was left to partners.

**Graham**: “I am fatalistic, I may have many ‘genes’.... BRCA didn't trouble me...I don't worry about things when I have no control or discomfort...I don't have breast cancer, I have a mutation...my wife does the telling”

In a few cases, family disharmony precluded disclosure, and this is found amongst both sexes.^24^, ^30^

**Stephen:** “I am not going to tell my 30 year old daughter about the breast...we don't speak to each other”

#### 3.2.8 Facing the Music: prostate cancer

Mutation status led to “facing the music” as men disclosed information to family members while remaining silent in respect of the ways it impinged on themselves. In contrast, subjective elements of PCa were conveyed, syphoned through constant descriptions of activity, good health, perspective, enabling self‐preservation.^9^, ^10^, ^13^, ^14^, ^15^, ^16^, ^29^

**Jeff**: “(then) prostate cancer!…I thought of myself as intelligent, good looking… girlfriends...now I've got hobbies and work and family...I'm just as good as before...written my first novel...live for the moment!”

Negative self‐perception when evoked did not deter men from elevating themselves by reporting “good health” and strength prior and post diagnosis in ways that suggested perspective, activity, control, rationality and statements of strength and “normality” all indicative of the ways that men reinstated their masculine selves.^10^

**George**: “Sexual function decreased ‐ not important ‐ I am interested in other things, fishing, motor sports...I'm alive...(I have) incontinence from the rear and front but there are people in wheelchairs...I thank my lucky stars that I'm unlike them.”

Restorative physical training found in other populations^10^, ^38^, ^39^ was not preferred amongst our participants. Finding new hobbies and being proactive were indicative of the ways that men restored “face” with optimism and fortitude.^10^, ^13^

**Nick: “**(......) I just go for it (cure for prostate cancer) I face it!”

A good relationship with the attending doctor and a need to gather information added to an already proactive stance.^7^

**Jeff**: “nerve wracking…getting (prostate) biopsy results...I phoned (doctor) in a state…she was always there for me. I became an expert...I was obsessed… it was my way of dealing with it... facing the music.”

## 4 DISCUSSION

A qualitative methodology has enabled us to “dig deep” into how men respond when facing two gendered conditions. By using masculinity theory, we have revealed dynamic and ambiguous responses that are sometimes similar to women in this context, challenging “innate” stereotypical ways of viewing men. Men present themselves as not only ideally masculine, but vulnerable too, sometimes simultaneously but always in ways that reinstate masculinity.

Our participants formed a homogenous group. Caution is advocated when working with diverse populations within and outside developed countries where “hegemonic masculinity” may be perceived in differing ways.^40^ Globalisation is changing the existence of gender orders in less developed countries, often mimicking Western versions of hegemonic masculinities.^40^ Where ambiguity exists, gender as a “relational” activity, occurring in diverse locations and differing contexts requires investigation.^28^, ^40^

Men with breast cancer respond in visceral ways.^20^, ^21^ Men in our sample experienced mutation status, possibly accounting for “silence” in the wake of an invisible disease. The gendering of breast cancer^20^, ^21^ and the lack of targeted information for men^20^, ^21^ may also have contributed to self‐confessed and relative “ignorance” concerning that condition. Like women, however,^17^ and men in other genetic studies,^17^, ^25^ we found men were mindful of families' wellbeing, especially that of daughters. Difficulties were articulated in relation to when and how to inform children in terms of both *BRCA* status and PCa.^17^ While men require help in that respect, reticence was balanced by an overwhelming need to inform genetic transmission to wider family members—countering the belief that men necessarily exclude themselves from health matters.^5^, ^9^ A “head of family” role is, however, indicative of the ways that men reinvent themselves as “masculine” in a context where stereotypical identities may be undermined.^10^

While women with *BRCA1/2* mutations^20^, ^21^ and men with PCa^10^, ^17^ do not generally exhibit clinical psychological symptoms, we were surprised that men were void of severe distress in the wake of a “double whammy,” seldom mentioned as a single entity—one that may have been too difficult to contemplate for all its gendered connotations, or because men were eager to put *BRCA* on a “back burner.” Men in our sample appreciated the bonus of early PCa diagnosis and the possibility of passing on knowledge regarding *BRCA1/2*. This may have allayed significant emotional fallout^17^ although serious psychological symptoms are not generally an issue for men with cancer and in larger samples than our own.^10^, ^13^, ^17^

By focusing on the treatment and aftermath of PCa, and by placing importance on an embodied disease, we suggest that a conduit was “produced” for normalising damaged male identities especially in the light of carrying a *BRCA1/2* mutation. As Wenger suggests, men with PCa are challenged “to manage a fully blown cancer that disrupts an embodied masculinity, the latter associated with action and strength.”^10^ Interestingly, sexual dysfunction was rarely evoked, unlike men in other studies,^14^, ^15^, ^16^ but nor was it a principle focus of our enquiry. Reticence may have relied on early stage PCa where nerve sparing prostatectomies preserve potency. Men did however exhibit distress in relation to both conditions.^10^, ^13^, ^17^, ^18^, ^20^, ^21^ Lives are changed, identity issues abound, responsibility and guilt towards families is stark, wrapped in stoicism, activity, positive self‐referral, knowledge building, and a need and appreciation of an “attachment” to health clinicians with whom they seemed to form a measure of equality and trust. In contrast to this, men shunned formal support.^10^

Men may wish to guard vulnerability while re‐establishing some “normality” and a sense of unscathed masculinity.^10^ This has to be respected but as Wenger says men may use help seeking such as knowledge gathering and eliciting the help of professionals^10^, ^35^ “to demonstrate power, control and even self‐reliance” as well as being “a possible indirect request for an intervention where men's help seeking is ‘socially problematic.’”^10^

There is an urgent need to research ways of providing support for men where physical^38^, ^39^ and/or informational resources and “good relationships” with health personnel^10^, ^35^ are addressed and where there is a recognition of men's gendered responses such as a need to maintain strength and identity^10^, ^38^, ^39^ *as well* as acknowledging so‐called feminine responses such as expression of emotion.^5^, ^8^ Men in our sample did not display overt emotion but underneath expressed bravado there lurked a palpable sense of sadness and fear.^17^

We do not believe that men (or women) necessarily experience specific and exclusive feelings when facing difficulty. The *ways* in which a man expresses distress may differ, usually to suit expectations for all the connotations of “masculinity” that we have alluded to. These caveats need to be cast in recognition of the pitfalls of essentialism as they lead to unequal care.

### 4.1 Clinical implications

Men appear to need help regarding disclosure to children especially boys; to understand *BRCA1/2* cancer risks and inheritance patterns; to access information and reassurance from clinicians, while being mindful of men's need to reinstate a sense of their masculine selves.

Research is required regarding support whilst recognising the possibility of differing needs in various populations of men. “Masculine behaviour may mask vulnerability to reinstate male identities and especially in the wake of bearing the “stigma” of having a so‐called “female” mutation as well as a full blown “masculine” cancer. Health professionals are asked to question their own gendered expectations, the latter possibly obfuscating men's experience, rendering invisible the “masculinities” that may be operating in this context and a need to recognise equal care.

### 4.2 Limitations

This study took place in one centre with a small homogeneous sample. More research using gender analyses is required that includes age, ethnicity, cultural diversity, sexuality, and socio‐economic status.

## Supporting information

Table S1: Interview Topics
Table S2: The sociodemographic characteristics of participants***
