# Supplementary material for: Ambiguity in a masculine world: Being a BRCA1/2 mutation carrier and a man with prostate cancer
Source: Psychooncology. 2017 Sep 18;26(11):1987–93. doi: 10.1002/pon.4530 (PMC5698714; doi:10.1002/pon.4530)
Supplement: Supplementary file 1 — Table S1: Interview Topics Table S2: The sociodemographic characteristics of participants*** [file PON-26-1987-s001.doc]

**Supplementary Table 1: Interview Topics**

| **Interview probes:**  **Reasons for testing/turning back on testing** |
| --- |
| Feelings regarding diagnosis of prostate cancer and mutation status |
| Impact of diagnosis / mutation on lifestyle |
| Impact on family including children |
| Sexuality |
| Impact on role as husband / father / breadwinner / worker |
| Information given at time of diagnosis |
| Communication issues |

|  | **Age at interview** | **Mutation**  **Status** | **Married/**  **Partner** | **Children** | **Children’s age(s)** | **Social**  **Class*** | **Age at prostate cancer diagnosis** | **Age mutation identified** | **Treatment** |
| --- | --- | --- | --- | --- | --- | --- | --- | --- | --- |
| Robert | 50 | *BRCA1+* | N/A | 2 F | 16,19 | II | 48 | 46 | Prostatectomy |
| Nigel | 53 | *BRCA2+* | N/A | 0 | - | II | 46 | 48 | Prostatectomy and Radiotherapy |
| Graham | 64 | *BRCA2+* | Y | 2 F | 34,37 | I | 57 | 53 | Radiotherapy |
| Stephen | 62 | *BRCA2+* | Y | 1 M  2 F | 19, 28,30 | I | 60 | 59 | Radiotherapy |
| Martin** | 65 | *BRCA2+* | Y | 1 M  1 F | 28,32 | II | 53 | 52 | Prostatectomy |
| Roger** | 71 | *BRCA1+* | Y | 2 F | 32,34 | I | 65 | 64 | Prostatectomy |
| Jeff | 46 | *BRCA2+* | Y | 1 M  1 F | 8,10 | II | 42 | 38 | Prostatectomy |
| Nick | 57 | *BRCA2+* | Y | 2 F  1M | 23,25,28 | III | 43 | 41 | Prostatectomy |
| George | 55 | *BRCA2+* | Y | 1 F | 22 | II | 54 | 52 | Prostatectomy |
| Edward | 55 | *BRCA1+* | Y | 1 M | 22 | II | 54 | 54 | Prostatectomy |
| Rupert | 62 | *BRCA2+* | Y | 1 M  1 F | 27,32 | II | 61 | 55 | Prostatectomy |
| Richard | 68 | *BRCA2+* | Y | 2 F | 36,38 | I | 65 | 61 | Prostatectomy |

**Supplementary Table 2:** **The sociodemographic characteristics of participants*****

*** The UK National Statistics Socio-economic Classification system was used**

** interviewed husband and wife together

*** The information in this table has been modified to preserve anonymity
